# Hierarchical control of bacterial growth efficiency by substrate and taxonomy

**DOI:** 10.64898/2026.07.26.740805

**Published:** 2026-07-29

**Authors:** Vaibhhav Sinha, Seppe Kuehn

## Abstract

Heterotrophic bacteria utilize organic carbon as a source of energy and material for growth. The balanced allocation of carbon between these two processes, termed growth efficiency, is a key physiological property of microbes because it links energy and biomass production, and determines the fraction of carbon lost as CO _2_ . However, we do not understand what controls growth efficiency in microbes or how it correlates with taxonomy and resource identity. Here, we develop a quantitative high-throughput method for measuring CO _2_ production during bacterial growth. For 23 bacterial strains spanning three phyla, grown on two carbon substrates, we quantify growth efficiency by measuring dynamic CO _2_ production and carbon accumulation in biomass. Intra-phylum comparisons show that glycolytic substrates yield higher efficiency growth (less CO _2_ produced per biomass carbon) than gluconeogenic substrates. However, growth efficiency varies as much across resources as it does across phyla. A physiological model shows that variation in the growth efficiency depends on the ATP produced per respired CO _2_ and the ATP needed per biomass on a given substrate, suggesting phylum level differences in energy supply and demand dictate differences in growth efficiency. This theory predicts no global correlation between growth rate and growth efficiency, a finding our data support. Finally, we report phylum-level variation in the dynamics of CO _2_ production, which we link to the presence of overflow metabolism on glycolytic sub-strates. This study revises our understanding of how taxonomy and substrate identity impact carbon allocation during growth and sets the stage for understanding CO _2_ production in communities.

**Significance:**

Bacteria are responsible for roughly half of all biological CO _2_ production on the planet, much of it in soils. How organic carbon flows through microbial metabolism sets the rate at which soils return CO _2_ to the atmosphere. Bacterial growth efficiency, defined by the fraction of consumed carbon that is retained as biomass rather than released as CO _2_ , determines the fluxes of carbon into the atmosphere. At present, we do not understand what controls growth efficiency in bacteria. We develop a new quantitative measurement of growth efficiency which we apply to 23 soil bacteria from three phyla on two substrates. We show that efficiency is structured hierarchically, at the highest level substrate identity defines efficiency which then varies substantially across taxa. So both the identity of species involved and the compounds they consume determine the rate of carbon loss.

## Full Text Availability

The license terms selected by the author(s) for this preprint version do not permit archiving in PMC. The full text is available from the preprint server.

